# Secondary traumatic stress in working nurses studying part time in a bachelor or Master’s nursing program in Croatia: a cross-sectional study

**DOI:** 10.1186/s12912-023-01691-1

**Published:** 2024-01-05

**Authors:** Marta Civljak, Ines Stivic, Livia Puljak

**Affiliations:** Center for Evidence-based Medicine and Health Care, Catholic University of Croatia, Ilica 242, Zagreb, 10000 Croatia

**Keywords:** Stress disorder, Secondary traumatic stress, Nursing, Occupational stress

## Abstract

**Background:**

Nurses are more likely to be exposed to human suffering than other healthcare professionals. Persons exposed to indirect trauma can experience symptoms of posttraumatic stress disorder, symptoms of avoidance, arousal and intrusion. Secondary traumatic stress (STS) occurs when a person hears about the firsthand traumatic experiences of another. This study aimed to explore the prevalence of STS among working nurses enrolled at a university nursing program.

**Methods:**

A cross-sectional study was conducted among nurses working in healthcare enrolled in university bachelor’s or Master’s nursing studies at the Catholic University of Croatia in November 2017. Data were collected using the *Secondary Traumatic Stress Scale* (STSS) and two items from the *World Health Organization* quality of life brief version (WHOQOL-BREF).

**Results:**

The study included 151 students; the response rate was 70%. The mean STS score was 38, indicating that the students on average suffered from moderate STS. Half of participating nurses met the criteria for STS. Working nurses enrolled in Master’s studies had lower STS scores than those enrolled into bachelor studies (t = 4.14, df = 149, p < 0.01). The level of STS had a negative correlation with participants’ quality of life assessment (r=-0.392, p < 0.01) and satisfaction with their health (r=-0.387, p < 0.01). We also found a significant positive correlation between subjective assessment of quality of life and satisfaction with personal health (r = 0.432, p < 0.01). We did not find a significant association between the level of STS and sex (r=-0.094) or years of nursing work experience (r=-0.069). Level of STS varied depending on the participants’ workplace, years of experience in that workplace and their work shifts. The highest levels of STS were seen in nurses working in the internal medicine department, those with 10–14 years of work experience in the current workplace, and those who work block shifts (12-hr shift followed by 24-hour shift).

**Conclusion:**

Over half of working nurses attending university studies had at least moderate STS. Furthermore, STS was negatively associated with participants’ perception of quality of life and satisfaction with their health. Prevention and alleviation interventions could reduce the burden of STS among nurses.

**Supplementary Information:**

The online version contains supplementary material available at 10.1186/s12912-023-01691-1.

## Background

Posttraumatic stress disorder (PTSD) was relatively recently added to the Diagnostic and Statistical Manual of Mental Disorders (DSM)– in 1980, after veterans of the Vietnam War were diagnosed with the disorder [1]. Since then, the effects of direct exposure to extreme traumatic stressors have been well explored, but it has also emerged that PTSD can develop in individuals who were indirectly affected by their close contact with a traumatized individual [1]. It has been observed that the negative effect of secondary exposure to extreme trauma can be the same as experienced by individuals who had primary exposure to such trauma [2].

Consequences of work-related indirect exposure to traumatic events of others have been conceptualized using several terms, including secondary traumatic stress (STS). The theoretical framework proposed by Bride et al., captures STS as a construct that is clearly distinct from job burnout [3]. Symptoms of STS include intrusion, avoidance, negative alterations in cognitions and mood, and alterations in arousal and reactivity [4].

In the context of “secondary trauma”, it has been hypothesized that there is a cost of caring for healthcare providers (HCP) dealing with traumatized patients [5]. Therefore, the occurrence of STS can be seen as an occupational hazard for HCPs. STS has been reported in mental health professionals, child protection professionals, clergy, social workers, as well as workplace lay trauma counselors (as reviewed in: [1]). Four reasons have been identified as contributing to the development of STS, including empathy, experiencing traumatic events in their own lives, unresolved personal trauma activated by another person’s trauma, and the particular impact of children’s trauma [6].

Some HCPs do not prefer using the term STS. Instead, they prefer the term compassion fatigue, as it has been described as being more friendly [6]. The term compassion fatigue [7] was reportedly first used by a nurse. However, there are also opinions that these two terms should not be used interchangeably, as STS is characterized by the presence of symptoms of PTSD, while compassion fatigue is a consequence of working with many traumatized persons in combination with empathic orientation [6].

It has been reported that STS is prevalent in nurses working in different fields [1], with prevalence ranging from 25% in forensic nurses [8] to 78% in hospice nurses [9]. A study conducted in Scotland among nurses employed in emergency departments revealed that 75% displayed at least one symptom covered by the STS scope [10]. Among Irish nurses employed in emergency medical services, 64% fulfilled the STS requirements [11]. Similarly, 52% of Jordanian emergency nurses reported having high or severe STS [12]. According to other studies, STS symptoms are common among critical care and oncology nurses [13, 14]. The effects of STS include poor problem solving and decision-making, poor concentration, difficulty sleeping, intrusive thoughts about patients, irritability, fear for the future, and diminished activity level [15]. Consequently, due to the symptoms of STS, nurses’ productivity at work may be affected, causing safety concerns for patients and other healthcare workers [15].

Systematic review about STS in nurses reported the need to further study the phenomenon of STS in nurses [1]. Therefore, the aim of this study was to explore the prevalence of STS among working nurses enrolled at a university nursing degree program at a university in Croatia. Furthermore, we aimed to explore whether there is a difference in the level of STS between working nurses enrolled in bachelor’s and Master’s level, and whether there is an association between demographic and professional characteristics and STS in nurses.

## Methods

### Study design

This was a cross-sectional study conducted in November 2017 at the Catholic University of Croatia, in Zagreb, Croatia. The research team included a psychiatrist (MC), a registered nurse (IS) and a research methodologist (LP).

### Participants

Inclusion criteria were: working nurses employed in healthcare, studying in university bachelor’s or Master’s programs at the Department of Nursing of the Catholic University of Croatia. We invited to participate in the study all students enrolled in these programs, who were eligible with their work status. These individuals have previously completed nursing high school (vocational degree), which enables them to work as HCPs. By choosing students from this University, we opted for a pragmatic convenience sample. This setting was chosen because the study investigators were employed at the Catholic University of Croatia; thus, the choice of the population was a convenient one.

Exclusion criteria were: students without nursing work experience, and students who accepted to participate in the study, but did not complete all the questionnaires used in the study.

### Ethics

The study was approved by the Ethics Committee of the Catholic University of Croatia, Zagreb, Croatia. Participants signed an informed consent before they were formally included to participate in the study. The study was conducted in accordance with the institutional Codes of Ethics. All methods were performed in accordance with the relevant guidelines and regulations.

### Data collection

Potential participants were approached during university classes, with the approval of university teachers heading those classes. They received written information about the study and were invited to participate in the study. Individuals who accepted to participate in the study and signed informed consent received paper-based questionnaires. All questionnaires were administered in Croatian language.

### Instruments

We used the *Secondary Traumatic Stress Scale* (STSS), a 17-item self-report measure of secondary trauma described by Bride et al. in 2004 [3]. The authors reported that the scale was a valid and reliable instrument for measuring STS [3]. The STSS was designed to analyze the reactions of helping professionals who had experienced traumatic stress via their work with traumatized clients. In line with the definition of PTSD in DSM-IV, in the STSS, the STS was operationalized using the factors intrusion, avoidance and arousal. To enable rigorous assessment of STS, the instrument is worded with stressor-specific items that refer explicitly to “client exposure” as a traumatic stressor. The STSS has become a standard tool for assessing STS in helping professionals [16]. For the purpose of this study, STSS was translated into Croatian language. The translation was conducted in line with the guidelines for cross-cultural adaptation of self-report measures [17].

The 17 items of STSS are preceded by the introduction stating that the participants are asked to read the list of statements made by persons who have been impacted by their work with traumatized clients. Participants are asked to indicate how frequently those statements were true for them in the past seven days, and there is a note that the word “client” is used to “*indicate persons with whom you have been engaged in a helping relationship. You may substitute another noun that better represents your work such as consumer, patient, recipient, etc.*.“ [3]. The participants are expected to circle the number on a 5-item Likert scale ranging from 1 (Never) to 5 (Very often). The three scales of the STSS are the intrusion scale (items 2, 3, 6, 10 and 13), avoidance scale (items 1, 5, 7, 9, 12, 14 and 17) and arousal scale (items 4, 8, 11, 15 and 16) [3].

Scores for the full STSS (all items) and each of the subscales are obtained by summing the items assigned to each. It is considered that an STS item is endorsed if a participant has chosen responses „occasionally“, „often“ or „very often“ in the preceding seven days (3, 4 or 5 points on a Likert scale, respectively). Bride recommended three approaches to scoring the STSS; one of them is using the algorithm described by Bride, the second one is using the classification of individuals into categories and the third one is using a cut-off value with the value of 38 recommended by Bride [18]. In this study, we used scoring by categories, as described by Bride, whereas there are five categories: scores less than 28 are interpreted as little or no STS, scores 28 to 37 are interpreted as mild STS, scores 38 to 43 are interpreted as moderate STS, scores 44 to 48 are interpreted as high STS, and 49 and above are interpreted as severe STS [18].

Additionally, we used two items from the *World Health Organization Quality of Life Scale brief version* (WHOQOL-BREF) [19]. WHOQOL-BREF is an abbreviated version of the WHOQOL-100 quality of life assessment, and it has been shown as valid and reliable [20]. The two items used in this study specifically asked about 1) the individual’s overall perception of their health [„*How would you rate your quality of life*?“ (item G1)] and the individual’s overall perception of their quality of life [„*How satisfied are you with your health*?“ (item G4)]. Participants answered those questions on a 5-item Likert scale ranging from 1 (very poor) to 5 (very good) [19].

Participants also answered questions about sociodemographic and professional characteristics of participants, including sex, age, study type (bachelor or Master’s), study year, workplace, years of nursing work experience, length of employment at the current job, daily work hours, place of employment, number of children, marital status (Appendix 1).

### Statistical analysis

Data were analyzed using quantitative analysis. We used frequencies and percentages for descriptive data analysis. We used mean (M) and standard deviation (SD) to describe the age of participants and average levels of STS. We used a t-test to explore the association between STS and level of education. We assessed the association between STS and satisfaction with health, quality of life, sex and years of professional experience using correlation analysis. We used a statistical significance level of p < 0.05. Data were analyzed using SPSS statistical software (SPSS, Inc., Chicago, IL, USA).

### Raw data

Raw data collected within this study are published on Open Science Framework (link: https://osf.io/wys8f/).

## Results

Among 217 invited students, 158 (73%) participated, and 151 (70%) completed questionnaires fully. Seven participants that did not complete all the questionnaires were excluded from the study.

The majority of participants were women, with average age 35 years; the majority had more than 10 years of work experience in nursing. The majority were married, with two children. Most participants worked as nurses in surgical departments or intensive care units, and most worked in block shifts (Table 1).

**Table 1:** Characteristics of participants (N = 151)

Variables	Categories	Descriptive statistics	
		N	%
Sex	Women	143	94.71
	Men	8	5.29
Year and level of university education	1st year of bachelor nursing studies	30	19.87
	2nd year of bachelor nursing studies	32	21.19
	3rd year of bachelor nursing studies	17	11.26
	1st year of Master’s nursing studies	39	25.83
	2nd year of Master’s nursing studies	33	21.85
Years of nursing work experience	< 6 months	2	1.32
	6 months– 4 years	23	15.24
	5–9 years	18	11.92
	10–14 years	28	18.54
	15–20 years	36	23.84
	20 > years	44	29.14
Marital status	Married	85	56.29
	Single	27	17.88
	In relationship	25	16.56
	Extramarital cohabitation	8	5.30
	Divorced	6	3.97
Number of children	0	69	45.70
	1	24	15.89
	2	38	25.17
	3 or more	20	13.24
Work place	Intensive Care Unit	24	15.89
	Surgery (including gynecology)	29	19.21
	Internal medicine	22	14.57
	Pediatrics	15	9.93
	Psychiatry	17	11.26
	Day clinic	9	5.96
	Other departments	35	23.18
Daily work hours	Only morning shits	50	33.11
	Two shifts (morning and afternoon shift)	19	12.58
	Block shifts (whole day, and whole night)	67	44.37
	Morning shift + 24-hour shift	13	8.61
	Other work schedules	2	1.35
		**M**	**SD**
Age (years)		35.32	7.84

N = number of participants, %=percent of participants, M = mean, SD = standard deviation

Among all students, the mean STS score was 38 (SD: 11.2), indicating that the students, on average, had moderate STS. Table 2 indicates the level of STS in participants, shown per year of university education, starting from year 1, which is the first year of a three-year bachelor level to year 5, which is the second year of a two-year Master’s level. Students of all levels of bachelor study level had, on average, higher levels of STS compared to students of Master’s study level (Table 2). Students of bachelor’s nursing level (42 ± 11.7) on average had significantly higher levels of STS compared to Master’s level (34 ± 9.3) students (t = 4.14, df = 149, p < 0.01). Almost a third of participants described mild STS, while more than half of participants reported higher levels of STS– moderate, high or severe STS (Table 2). The most prevalent symptom in students of all levels was intrusive thoughts about clients (Table 3).

**Table 2:** Prevalence of secondary traumatic stress (STS) in participants at different levels of university studies (N = 151)

Levels of STS	Year of university studies					Total (N)
	1.	2.	3.	4.	5.	
Little or no STS	3	4	2	10	8	27 (18)
Mild STS	11	5	5	15	12	48 (32)
Moderate STS	6	6	4	7	9	32 (21)
High STS	4	6	2	7	0	19 (13)
Severe STS	6	11	4	0	4	25 (17)
M	39	43	42	33	35	
SD	9.7	13.1	11.7	8.9	9.8	

1 = 1st year of bachelor nursing studies, 2 = 2nd year of bachelor nursing studies, 3 = 3rd year of bachelor nursing studies, 4 = 1st year of Master’s nursing studies, 5 = 2nd year of Master’s nursing studies, N = number of participants, M = mean, SD = standard deviation

**Table 3:** Prevalence of symptoms of intrusion, avoidance and arousal per year of university studies expressed as mean score (N = 151)

	Name of the symptom	Year of university studies				
		1.	2.	3.	4.	5.
Symptoms of intrusion (M)	Intrusive thoughts about clients	3.10	3.38	3.24	2.69	2.70
	Disturbing dreams about clients	1.97	2.50	1.82	1.85	1.82
	Sense of relieving clients’ trauma	2.47	2.44	2.76	1.90	1.94
	Cued psychological distress	1.93	2.16	2.18	1.51	1.70
	Cued psychological reaction	2.07	2.16	2.59	1.67	1.70
Symptoms of avoidance (M)	Avoidance of clients	2.13	2.53	2.71	1.90	1.97
	Avoidance of people, places, things	1.70	2.34	1.88	1.38	1.58
	Inability to recall client information	1.90	2.03	2.35	1.54	1.79
	Diminished activity level	2.40	2.56	2.41	2.13	2.39
	Detachment from others	1.87	2.16	2.00	1.77	1.58
	Emotional numbing	2.83	3.06	2.47	2.28	2.52
	Foreshortened future	2.33	2.56	2.53	1.95	2.15
Symptoms of arousal (M)	Difficulty sleeping	2.63	2.78	2.35	1.97	1.94
	Irritability	2.50	2.84	2.47	2.08	2.18
	Difficulty concentrating	2.67	2.97	2.82	2.41	2.39
	Hypervigilance	2.03	2.50	2.47	1.90	2.00
	Easily startled	2.63	2.88	2.65	2.44	2.55

1 = 1st year of bachelor nursing studies, 2 = 2nd year of bachelor nursing studies, 3 = 3rd year of bachelor nursing studies, 4 = 1st year of Master’s nursing studies, 5 = 2nd year of Master’s nursing studies, M = mean

Analysis of the association between symptoms of STS and different participants’ characteristics indicated that the level of STS had a negative correlation with subjective assessment of their quality of life (r=-0.392, p < 0.01); this negative correlation indicates that participants with lower levels of STS have assessed their quality of life higher. A negative correlation was found between STS and satisfaction with their health (r=-0.387, p < 0.01), indicating that participants with lower STS levels were more satisfied with their health. We also found a significant positive correlation between subjective assessment of quality of life and satisfaction with personal health (r = 0.432, p < 0.01). We did not find a significant association between the level of STS and sex (r=-0.094) or years of nursing work experience (r=-0.069).

Level of STS varied depending on the participants’ workplace, years of experience in that workplace and their work shifts. The highest levels of STS were seen in nurses working in the internal medicine department, those with 10–14 years of work experience in the current workplace, and those who work block shifts (Table 4).

**Table 4:** Levels of secondary traumatic stress (STS) in participants with different work place, years of experience on that work place and work shifts (N = 151)

		Level of STS (N)						
		Little or no STS	Mild STS	Moderate STS	High STS	Severe STS	M	SD
Work place	Intensive care unit	3	10	5	1	5	39	13.1
	Surgery	2	11	9	5	2	38	8.1
	Internal medicine	2	5	5	4	6	42	10.7
	Pediatrics	5	4	1	1	4	36	16.1
	Psychiatry	6	5	3	2	1	33	9.6
	Day clinic	2	2	2	2	1	40	10.1
	Other	7	11	7	4	6	36	10.5
Years working in that place	< 6 months	2	1	2	1	1	38	8.7
	6 months– 4 years	13	12	8	6	7	37	12.2
	5–9 years	4	9	7	3	2	36	9.4
	10–14 years	1	10	6	4	8	42	9.9
	15–20 years	4	9	5	2	3	36	10.3
	20 > years	3	7	4	3	4	41	13.4
Work shifts	Only morning shits	7	22	6	6	9	38	11.4
	Two shifts (morning and afternoon shift)	5	6	3	5	0	35	10.3
	Block shifts (whole day, and whole night)	11	17	18	6	15	40	11.3
	Morning shift + 24-hour shift	3	2	5	2	1	37	9.9
	Other work schedules	1	1	0	0	0	24	3.8

N = number of participants, M = mean, SD = standard deviation

## Discussion

The main finding of this study is that more than half of the working nurses enrolled into a university nursing degree program had at least moderate levels of secondary traumatic stress. This is in line with previous results of Beck et al., who reported in 2017 that 49% of nurses employed at neonatal intensive care units (sample size: N = 175) had at least moderate scores of STS [21]. In 2015, Duffy et al. showed that 64% of emergency nurses (sample size: N = 117) met the criteria for secondary traumatic stress [11]. Nicholls et al. explored STS among labor and delivery nurses (sample size: N = 144), and reported that 35% had at least moderate STS levels [22]. A study published in 2013 that analyzed nurses in Iran (sample size: N = 173) showed that 40% of nurses had symptoms of STS [23]. All these studies indicate that STS is highly prevalent among nurses.

With regard to marital status, all groups of participants reported moderate STS on average, except for those in a relationship where STS was slightly lower on average and corresponded to a mild level. In a study on female nursing students in Kenya, widowed/separated/divorced participants had higher scores on the STSS than married and single nurses [24]. A study on nurses from Iran showed an association between a lack of social support from a significant other, family and friends and STS [23].

In our study, STS levels were lower in nurses attending Master’s study program. This was in line with the study published by Mangoulia et al. in Greece, which showed that participants with lower levels of education had a more pronounced STS/compassion fatigue [25]. The authors suggested that education could be important because education is considered a protective effect, and because nurses with higher education have less contact with patients, as their work also includes managerial tasks [25]. In a sample of 221 adult, pediatric, and neonatal critical care nurses in the USA, Sacco et al. have also shown that nurses with higher education levels have lower STS [26]. Townsend and Campbell have shown the same in a study which included sexual assault nurse examiners [8].

Contrary results were published in 2013 by Michalec et al., who analyzed burnout and compassion fatigue among 436 undergraduate nursing students [27]. Their study found no difference between 3rd and 4th -year students and those of the 1st or 2nd -year students. Nursing students reported experiencing emotional exhaustion, depersonalization, burnout, and STS but only at low and moderate/average levels. However, as noted by Michalec et al., the role of the student, compared to a professional nurse, could provide a “safety net” in terms of patient responsibility and the effects on well-being that accompany such responsibilities. The authors thus wondered whether their results indicated “*the calm before the storm*” [27].

In our sample, we included only nursing students that were employed as nurses in the healthcare system. Thus, our participants should not be considered as those who are shielded with a university safety net because they are experiencing the stresses of the healthcare profession daily.

We found differences among nurses based on their workplace. The highest STS was observed among nurses working in the internal medicine departments. However, there are various subtypes of internal medicine specialties and it would be interesting to examine further whether there are any differences in STS between nurses working in various internal medicine departments based on subspecialties.

The most common symptom suffered by all participants in our study was intrusive thoughts about clients. A study among oncological nurses indicated that intrusive thoughts about clients were among the most commonly described symptoms of the participants [28]. Emotional numbing was the most common symptom of avoidance in our study. This finding was in line with the study of Duffy et al. that was conducted among emergency nurses [11].

Nurses with 10 to 14 years of workplace experience reported the highest STS levels. A study of Wu et al., conducted among American and Canadian oncology nurses, reported that nurses aged 40 years and less had a higher risk of developing compassion fatigue [29]. Older age combined with more years of workplace experience was shown as a protective factor against STS. Perhaps this could also be explained by the differences in work tasks between younger and older nurses.

In this study, nurses who worked block shifts reported the highest STS levels. This was in line with the study of Hinderer et al., conducted among trauma nurses, where working longer shifts was associated with higher compassion fatigue [30].

Healthy and supportive work environments are crucial for nurses’ health, well-being, and satisfaction. Interventions that would lead to improvements in the workplace could help prevent negative consequences and improve health outcomes for patients and nurses, decrease nurse turnover, and reduce healthcare expenditures. The findings of this study can be used for implementing changes in healthcare institutions, such as developing preventive interventions and psychosocial support for nurses [29].

In 2022, Robinson et al. reported the results of developing and implementing a 5-week secondary traumatic stress reduction program in a pilot sample of nine emergency department nurses. Their findings indicated that the program led to a significant reduction in secondary traumatic stress and related somatic symptoms [31].

Recommendations and implications for institutions and policymakers include considering interventions for prevention of STS. Suggested preventive interventions for STS include providing training for raising awareness of STS, offering opportunities for employees to explore their trauma histories, supporting reflective supervision in which a healthcare worker and a supervisor regularly meet to address feeling regarding interactions with patients [32].

This study had some limitations. We used pragmatic convenience sampling, which may limit the generalizability of the findings because of non-response bias. The participants were self-selected based on their availability and willingness to participate. It is possible that non-responders were meaningfully different than responders, which can affect study findings. Of note, our study had a high response rate (70%), which, hopefully, minimizes the non-response bias. It has been postulated that non-response bias becomes critical with response rates below 70% [33]. Also, the sample was limited in terms of encompassing nurses studying at one university, a single institution in Croatia. This sample may not represent the broader population of nurses both in Croatia and globally. The lack of diversity in the sample (in terms of location, type of institution, and cultural factors) could impact the generalizability of the findings. Of note, included nurses were heterogeneous in terms of workplaces and other personal characteristics. Moreover, due to sensitive nature of the topic, there is a possibility that the participants might have underreported or overreported their experiences. This was mitigated with anonymous nature of the study.

We did not conduct sample size calculation. Instead, we invited all eligible students to participate in the study. Furthermore, this was a cross-sectional study. A longitudinal study would allow a better interpretation of the development and maintenance of secondary trauma symptoms in nurses. Also, history of mental illness puts a person at greater risk of STS. In our study, we did not control for the potential effect of the history of mental illness.

In conclusion, over half of working nurses attending university studies had at least moderate STS. Furthermore, STS was negatively associated with participants’ perception of quality of life and satisfaction with their health. Prevention and alleviation interventions could reduce the burden of STS among nurses. Healthcare institutions and policymakers should prioritize mental health of healthcare workers by engaging in preventive strategies.

### Electronic supplementary material

Below is the link to the electronic supplementary material.

**Supplementary Material 1:** Questionnaire about sociodemographic and professional characteristics of participants

## Data Availability

Raw data collected within this study are published on Open Science Framework (link: https://osf.io/wys8f/).

## Acronyms

STS: Secondary traumatic stress
STSS: Secondary traumatic stress scale
PTSD: Posttraumatic stress disorder
DSM: Diagnostic and Statistical Manual of Mental Disorders
WHOQOL-BREF: World Health Organization quality of life brief version
