# Supplementary material for: Secondary traumatic stress in working nurses studying part time in a bachelor or Master’s nursing program in Croatia: a cross-sectional study
Source: BMC Nurs. 2024 Jan 5;23:22. doi: 10.1186/s12912-023-01691-1 (PMC10768158; doi:10.1186/s12912-023-01691-1)
Supplement: Supplementary file 1 — Supplementary Material 1: Questionnaire about sociodemographic and professional characteristics of participants [file 12912_2023_1691_MOESM1_ESM.docx]

# Appendix 1. Questionnaire about sociodemographic and professional characteristics of participants

**1. Sex:**

a) woman

b) man

**2. Age? (years) ____________________**

**3. Marital status**

a) married

b) single

c) in relationship

d) extramarital cohabitation

e) divorced

f) widowed

**4. Number of children**

a) 0

b) 1

c) 2

d) 3 or more

**5. Year and level of university education**

a) 1^st^ year of bachelor nursing studies

b) 2^nd^ year of bachelor nursing studies

c) 3^rd^ year of bachelor nursing studies

d) 1^st^ year of Master’s nursing studies

e) 2^nd^ year of Master’s nursing studies

**6. Years of working nursing experience**

a) less than 6 months

b) 6 months to 4 years

c) 5 -9 years

d) 10-14 years

e) 15-20 years

f) > 20 years

**7. Years of work experience at the current work place**

a) less than 6 months

b) 6 months to 4 years

c) 5 -9 years

d) 10-14 years

e) 15-20 years

f) > 20 years

**7. Current work place**

a) Intensive care unit

b) Surgery (including gynecology)

c) Internal medicine

d) Pediatrics

e) Psychiatry

f) Day clinic

g) Other (please describe) ___________________

**8. Work shifts**

a) Only morning shift

b) Only afternoon shift

c) Only night shifts

d) Two shifts (morning and afternoon)

e) Block shifts (day and night)

f) Morning shift plus 24-hour shift

g) Other (please describe) ___________________
